# The differences in self-perceptions of aging, health-related quality of life and their association between urban and rural Chinese older hypertensive patients

**DOI:** 10.1186/s12955-020-01411-2

**Published:** 2020-05-26

**Authors:** Yunying Hou, Qing Wu, Dandan Zhang, Xiaohong Jin, Wenya Wu, Xiaohua Wang

**Affiliations:** 1 Department of Cardiology, the First Affiliated Hospital of Soochow University, Suzhou, 215006 China; 2 School of Nursing, Soochow University, Suzhou, 215006 China; 3 Quality Improvement Office, Changshu No.1 People’s Hospital, Changshu, 215500 China; 4 Department of Cardiology, Changshu No.1 People’s Hospital, Changshu, 215500 China

**Keywords:** Self-perceptions of aging, Health-related quality of life, Hypertension, Urban, Rural, Chinese

## Abstract

**Background:**

Most hypertensive clients are elderly, whose health-related quality of life (HRQL) may be associated with self-perceptions of aging (older individuals’ beliefs about their own aging). Meanwhile, culture and health disparities between rural and urban populations are substantial. Whether there are differences in self-perceptions of aging, HRQL, and their association among elderly hypertensive clients in urban and rural areas remains unknown. The objective of this study was to investigate and compare self-perceptions of aging and HRQL and their association among urban and rural older Chinese hypertensive clients.

**Methods:**

A cross-sectional investigation was conducted in 15 urban community clinics and 22 village clinics from Suzhou, China. Older hypertensive adults were invited to complete a self-administered questionnaire addressing socio-demographic and clinical information, HRQL and self-perceptions of aging.

**Results:**

There were 492 urban participants and 537 rural participants included in the analyses. The physical (40.0 ± 12.1 vs. 30.9 ± 8.9, *P* <  0.001) and mental (51.5 ± 8.3 vs. 46.0 ± 7.8, *P* <  0.001) HRQL scores of urban participants were all higher than those of rural ones. Urban participants’ scores on dimensions of “timeline cyclical”, “consequences negative”, and “control negative” of self-perceptions of aging questionnaire (APQ) were lower than those of rural participants (*P* <  0.001, respectively), while the scores on dimensions of “consequences positive” and “control positive” were higher (*P* <  0.001, respectively). Adjusted multivariate linear regression showed that participants who had worse self-perceptions of aging had poorer HRQL. Some APQ dimensions associated with urban or rural hypertensive elders’ HRQL were different.

**Conclusions:**

Older hypertensive clients in rural areas have poorer self-perceptions of aging and HRQL than those in urban areas. Health care professionals should pay more attention to HRQL and self-perceptions of aging of older hypertensive clients in rural areas.

## Background

The prevalence of hypertension is increasing [1], and most hypertensive patients are elderly. The prevalence of hypertension in older adults ≥65 years in Mainland China is as high as 56.51% [2]. Hypertension is a major risk factor for coronary artery disease, stroke and renal failure, and it is one of the major causes of disability and death in the elderly [3]. Hypertensive older clients have more healthcare needs and are more likely to have poorer health-related quality of life (HRQL) than normotensive elderly people [4]. However, intensive treatment of blood pressure (BP) did not significantly affect HRQL [5]. Therefore, it is necessary to explore other factors associated with older hypertensive clients’ poorer HRQL.

One of the factors that may be associated with quality of life is self-perceptions of aging [6, 7]. Self-perceptions of aging were found to have a significant longitudinal impact on health and health behaviors in a meta-analysis of 19 studies [8]. Adults who have more positive self-perceptions of aging are more likely to engage in positive life style and have good psychological resources even in old age [9–13]. Older people who have the belief that health problems are inevitable in old age are unwilling to use preventive health service, and are more likely to have a heightened cardiovascular response to stress [14]. In contrast, poor health may lead to negative self-perceptions of aging [15, 16]. Given the high prevalence of hypertension, and the possible positive change in self-perceptions of aging among older adults [17], promoting positive self-perceptions of aging may modify the poor HRQL of hypertensive older adults. However, the association between self-perceptions of aging and HRQL among older hypertensive people has not been thoroughly explored. Meanwhile, culture, thought and ideas, and health disparities between rural and urban populations have grown substantially with the process of rapid urbanization over past decades [18, 19]. Whether there are differences in self-perceptions of aging, HRQL, and their association among elderly hypertensive clients in urban and rural areas remains unknown. The objective of the present study was to investigate and compare self-perceptions of aging and HRQL and their association among urban and rural older Chinese hypertensive clients.

## Methodology

This study is a cross-sectional investigation, and information was gathered primarily through self-administered questionnaires, including basic socio-demographic and clinical data, self-reported HRQL, and self-perceptions of aging. Socio-demographic and clinical data included age, gender, marital status, educational attainments, living arrangements, status of BP control, duration of hypertension, the number of comorbidities and body mass index (BMI), which were covariates of HRQL and must be controlled when analyzing the association between self-perceptions of aging and HRQL [20]. After obtaining signed informed consent forms, investigators invited patients to complete the investigation. Nurse and student investigators were trained in questionnaire administration before the start of the survey, including data collection and verification methods, and how to communicate accurately and effectively with participants. For illiterate participants, investigator researchers read the question items word-by-word, exactly as printed on the questionnaire. At the end of the investigation, each participant had his or her BMI measured. This study was approved by the ethics committee of Changshu No.1 People’s Hospital.

### Study population

This cross-sectional survey was carried out in 15 urban community clinics and 22 rural village clinics in Suzhou, China. Because the population density of Suzhou municipality is less than 1500 people per square kilometer, we defined the areas where the sub-district offices were located and the constructed towns as urban, and the other areas where more than 50% residential population were farmers as rural, according to the demarcation standards of the National Bureau of Statistics and the State Council of China. There were approximately 3, 800 inhabitants in each urban community and 4, 800 inhabitants in each rural village. Participants aged 60 years or above who sought medical advice in study clinics were recruited between November 2013 and December 2016. Subjects who reported ever having been diagnosed with hypertension by a qualified health care provider and were able to communicate were invited to participate in this study. Hypertension was defined as systolic blood pressure (SBP) ≥ 140 mmHg and/or diastolic blood pressure (DBP) ≥ 90 mmHg or the use of antihypertensive medications [21]. Each potential subject was verbally asked about the history of their diseases and medications and verified by their medical records to confirm study eligibility. Subjects were excluded if they had any of the following conditions that would prohibit participation or have a great impact on HRQL: dementia or cognitive impairment, cancer, heart failure (New York Heart Association functional class III or IV), or unstable angina.

We used G^*^Power version 3.1 software to estimate the sample size. With a medium effect size at 0.10 and a power of 0.90, the sample size for 16 predictors to achieve α at 0.01 was 331. Considering that there might be 30% of questionnaires having missing or implausible information, the total sample size of urban and rural hypertensive patients should not be less than 860.

### Instrument

The participants’ age, gender, marital status, educational attainments, living arrangements, status of BP control, duration of hypertension, and the number of comorbidities were gathered through a self-reported survey by trained researchers. Hypertensive clients with BP < 140/90 mmHg in their last appointment were considered to have their BP controlled. The height and weight were measured using a calibrated stadiometer and weight scale for patients wearing light clothing without shoes. The BMI was calculated as the weight (in kilograms) divided by the square of the height (in metres).

HRQL was measured using the short form-36 (SF-36), which has been used in different populations [22–24]. SF-36 is a self-administered questionnaire that generates assessment scores across 36 scales. These scales are scored on a 0–100 scale, with higher scores indicating better HRQL. The following 8 dimensions of HRQL are evaluated by these scores: physical functioning (PF), role limitations due to physical problems (RP), bodily pain (BP), general health perceptions (GH), vitality (VT), social functioning (SF), role limitations due to emotional problems (RE), and mental health (MH). The SF-36 dimensions can be reduced to 2 aggregate summaries with the first four dimensions indicating respondents’ physical component summary (PCS) and the last four indicating mental component summary (MCS) [25]. The mandarin version of the SF-36 used in this study has been administered successfully in general populations in China with the internal reliability coefficients of the PCS and MCS scales ranging from 0.85 to 0.87 [26, 27] and its validity and reliability have been tested in Chinese hypertensive clients [28]. The PCS and MCS scores were calculated based on the Chinese population norm [27].

Self-reported perceptions of aging were measured using the 32-item aging perceptions questionnaire (APQ) developed by Barker et al. in 2007 [29]. The 32 items comprised 7 subscales examining aging views: timeline chronic, timeline cyclical, consequences positive, consequences negative, control positive, control negative, and emotional representations. “Timeline chronic” relates to the awareness of the chronic nature of aging (e.g., ‘I always classify myself as old’), and “timeline cyclical” reflects the variation in the awareness of aging (e.g., ‘I go through phases of feeling old’). “Consequences” refers to beliefs about the impact of aging on one’s life, including positive consequences (e.g., ‘As I get older, I get wiser’) and negative consequences (e.g., ‘Getting older makes everything harder for me’). “Control” refers to elders’ beliefs about managing one’s experience of aging, which can be positive (e.g., ‘The quality of my social life in later years depends on me’) or negative (e.g., ‘Immobility in later life is not up to me’). “Emotional representations” demonstrate the negative emotions generated by aging (e.g. ‘I get depressed when I think about getting older’). Answers are provided on a 5-point scale, ranging from 1 ‘strongly disagree’ to 5 ‘strongly agree’. The response scale of the control negative dimension is reversed (1 ‘strongly agree’ to 5 ‘strongly disagree’). The mean score for each subscale is calculated with higher scores indicating greater endorsement of a specific perception. A Chinese version of the APQ was used, with Cronbach alpha coefficients of 0.884 for the total scale, 0.869 for timeline chronic, 0.700 for timeline cyclical, 0.665 for consequences positive, 0.836 for consequences negative, 0.822 for control positive, 0.748 for control negative, 0.835 for emotional representations, and cumulative variance contribution rate 65.269% [30].

### Analytical strategy

All data were analyzed using SPSS 20.0 (SPSS, Chicago, Illinois, USA). All continuous variables were expressed as mean ± standard deviation (SD), and the categorical variables were summarized by frequencies or percentage. Socio-demographic and clinical characteristics were calculated for rural and urban participants, and the significance of differences across groupings were determined using the Chi-square test for nominal variables and Student’s *t*-test for independent samples for continuous variables (Table 1). Multivariate analysis of variance (MANOVA) was used to control demographic characteristics and compare the differences of HRQL and self-perceptions of aging between urban and rural participants (Tables 2 and 3). Multivariate linear regression was utilized to assess the association between self-perceptions of aging and PCS and MCS adjusted for age (numerical), gender, marital status (single or divorced or widowed vs. married), educational attainments (junior middle school and below vs. senior high school and above), living arrangements (with family vs. alone), BMI (numerical), duration of hypertension (numerical), and comorbidities (with vs. without) (Tables 4 and 5). The coefficient and 95% confidence interval (95% CI) are presented. All statistical tests were two-sided and tests of alpha less than 0.05 were considered statistically significant.

**Table 1:** Socio-demographic and Clinical Characteristics for Urban and Rural Participants

Variable	Urban participants (*n* = 492)	Rural participants (*n* = 537)	*x*^*2*^/*t*	*P*
Gender, *n* (%)			4.890	0.027
Male	263 (53.5%)	250 (46.6%)		
Female	229 (46.5%)	287 (53.4%)		
Age (years), *Mean ± SD*	68.4 ± 7.5	70.8 ± 9.2	−4.569	< 0.001
Marital status, *n* (%)			4.968	0.026
Single/divorced/widowed	91 (18.5%)	130 (24.2%)		
Married	401 (81.5%)	407 (75.8%)		
Educational attainments, *n* (%)			196.371	< 0.001
Junior middle school and below	316 (64.2%)	526 (98.0%)		
Senior high school and above	176 (35.8%)	11 (2.0%)		
Living arrangements, *n* (%)			3.342	0.068
Alone	44 (8.9%)	32 (6.0%)		
With family	448 (91.1%)	505 (94.0%)		
BMI (kg/m^2^), *M* ± *SD*	23.6 ± 3.2	24.0 ± 4.0	−1.845	0.065
Blood pressure control, *n* (%)			158.759	< 0.001
Yes	366 (74.4%)	189 (35.2%)		
No	126 (25.6%)	348 (64.8%)		
Hypertension duration (years), *Mean* ± *SD*	9.1 ± 8.3	10.8 ± 7.2	−3.480	0.001
Comorbidities, *n* (%)			0.173	0.677
No	263 (53.5%)	294 (54.7%)		
Yes	229 (46.5%)	243 (45.3%)		

*Note*. *SD* standard deviation, *BMI* body mass index

**Table 2:** SF-36 Scores for Urban and Rural Participants

Domain, *M*ean ± *SD*	Urban participants (*n* = 492)	Rural participants (*n* = 537)	*F*	*P*
Physical functioning	73.9 ± 22.5	70.5 ± 23.9	15.384	< 0.001
Role physical	65.2 ± 38.0	80.2 ± 36.7	10.808	0.001
Bodily pain	76.6 ± 20.5	9.1 ± 13.0	1.017E3	< 0.001
General health	44.1 ± 16.3	45.8 ± 6.2	0.618	0.432
Vitality	67.8 ± 16.5	59.6 ± 14.8	112.239	< 0.001
Social functioning	85.4 ± 16.4	45.7 ± 20.3	285.632	< 0.001
Role emotional	69.7 ± 37.2	85.4 ± 31.8	10.231	0.001
Mental health	65.6 ± 15.8	57.9 ± 14.8	50.085	< 0.001
PCS	40.0 ± 12.1	30.9 ± 8.9	144.300	< 0.001
MCS	51.5 ± 8.3	46.0 ± 7.8	136.752	< 0.001

*Note*. *SD* standard deviation, *SF-36* 36-item short-form health survey, *PCS* physical component summary, *MCS* mental component summary

**Table 3:** Scores on Each Domain of Self-perceptions of Aging for Urban and Rural Participants

Domain, *Mean* ± *SD*	Urban participants (*n* = 492)	Rural participants (*n* = 537)	*F*	*P*
Timeline chronic	3.0 ± 0.9	3.1 ± 0.8	0.017	0.897
Timeline cyclical	3.3 ± 0.7	3.6 ± 0.6	32.923	< 0.001
Consequences positive	3.5 ± 0.7	3.3 ± 0.7	17.493	< 0.001
Consequences negative	3.1 ± 0.8	3.6 ± 0.6	114.586	< 0.001
Control positive	3.6 ± 0.7	3.3 ± 0.6	45.438	< 0.001
Control negative	3.0 ± 0.7	3.4 ± 0.6	19.993	< 0.001
Emotional representations	2.7 ± 0.8	2.6 ± 0.6	0.428	0.513

*Note*. *SD* standard deviation

**Table 4:** Association between Self-perceptions of Aging and PCS for Urban and Rural Participants

	Urban participants (*n* = 492)			Rural participants (*n* = 537)		
Variable	Coefficient	95% *CI*	*P*	Coefficient	95% *CI*	*P*
Self-perceptions of aging						
Timeline chronic	−1.17	−2.28, − 0.06	0.038	−3.21	−4.24, − 2.18	< 0.001
Timeline cyclical	− 0.01	− 1.66, 1.64	0.988	− 1.91	− 3.64, − 0.18	0.031
Consequences positive	− 0.19	−1.54, 1.16	0.779	1.15	0.07, 2.24	0.037
Consequences negative	−2.07	−3.43, − 0.71	0.003	1.57	− 0.21, 3.35	0.084
Control positive	2.08	0.70, 3.45	0.003	0.34	−0.92, 1.60	0.596
Control negative	0.26	−1.20, 1.72	0.724	− 1.45	−3.03, 0.13	0.073
Emotional representations	−1.50	−2.97, −0.03	0.045	−4.31	−5.56, −3.05	< 0.001
Controlled variables						
Gender (ref: male)	−2.36	−4.22, −0.49	0.013	− 1.81	−3.16, − 0.46	0.009
Age	−0.20	− 0.35, − 0.06	0.006	−0.07	− 0.17, 0.03	0.152
Marital status (ref: single/divorced/widowed)	2.61	−0.17, 5.38	0.065	0.43	−1.35, 2.22	0.663
Educational attainments (ref: junior middle school and below)	−0.53	−2.49, 1.43	0.598	−4.43	−9.16, 0.29	0.066
Living arrangements (ref: alone)	−2.15	−5.78, 1.48	0.246	−1.84	−4.89, 1.21	0.236
BMI	−0.04	−0.32, 0.24	0.789	0.10	−0.07, 0.27	0.254
Blood pressure control (ref: no)	0.68	−1.38, 2.73	0.519	0.16	−1.26, 1.56	0.830
Hypertension duration	−0.10	−0.22, 0.02	0.111	−0.09	−0.20, 0.01	0.069
Comorbidities (ref: no)	−7.34	−9.35, −5.33	< 0.001	−0.15	−1.60, 1.29	0.834

*Note*. *CI* confidence interval, *PCS* physical component summary, *BMI* body mass index

**Table 5:** Association between Self-perceptions of Aging and MCS for Urban and Rural Participants

	Urban participants (*n* = 492)			Rural participants (*n* = 537)		
Variable	Coefficient	95% *CI*	*P*	Coefficient	95% *CI*	*P*
Self-perceptions of aging						
Timeline chronic	0.11	−0.75,0.97	0.801	−2.52	−3.47, −1.56	< 0.001
Timeline cyclical	−0.86	−2.14, 0.43	0.191	0.17	−1.45, 1.78	0.841
Consequences positive	0.45	−0.60, 1.50	0.399	−0.25	−1.25, 0.76	0.632
Consequences negative	−0.04	−1.10, 1.01	0.935	−1.13	−2.79, 0.52	0.179
Control positive	1.89	0.82, 2.96	0.001	0.62	−0.55, 1.78	0.302
Control negative	0.94	−0.20, 2.07	0.106	1.43	−0.04, 2.91	0.057
Emotional representations	−2.41	−3.55, −1.26	< 0.001	−1.72	−2.88, −0.55	0.004
Controlled variables						
Gender (ref: male)	1.32	−0.13, 2.77	0.074	1.57	0.31, 2.83	0.015
Age	0.20	0.09, 0.31	0.001	−0.02	−0.12, 0.07	0.632
Marital status (ref: single/divorced/widowed)	−0.42	−2.58, 1.73	0.701	−1.64	−3.30, 0.02	0.053
Educational attainments (ref: junior middle school and below)	0.49	−1.03, 2.02	0.526	−1.11	−5.51, 3.28	0.620
Living arrangements (ref: alone)	1.19	−1.63, 4.02	0.407	1.18	−1.66, 4.01	0.416
BMI	0.18	−0.04, 0.40	0.101	−0.10	− 0.26, 0.06	0.210
Blood pressure control (ref: no)	0.26	−1.34, 1.86	0.752	−0.22	−1.54, 1.09	0.738
Hypertension duration	0.04	−0.06, 0.13	0.435	0.05	−0.05, 0.14	0.331
Comorbidities (ref: no)	−2.65	−4.22, −1.09	0.001	0.51	−0.84, 1.85	0.458

*Note*. *CI* confidence interval, *MCS* mental component summary, *BMI* body mass index

## Results

### Participant characteristics

A total of 1068 copies of the initial questionnaires were collected. Among those collected, 39 (3.7%) were excluded due to missing data or implausible data, including persons who did not have a blood pressure measurement (*n* = 7) and those who did not complete the SF-36 HRQL or APQ or who chose the same score on each item when filling out APQ (*n* = 32). Finally, there were 492 urban participants and 537 rural participants included in the analyses. Table 1 presents the socio-demographic and clinical characteristics. Compared with participants who lived in rural areas, more participants living in urban areas were men (53.5% vs. 46.6%, *P* = 0.027), younger (68.4 ± 7.5 vs. 70.8 ± 9.2 years old, *P* <  0.001), married (81.5% vs. 75.8%, *P* = 0.026), had a higher education level (35.8% vs. 2.0% had an education higher than senior high school, *P* <  0.001) and shorter hypertension duration (9.1 ± 8.3 vs. 10.8 ± 7.2 years, *P* = 0.001), and had their blood pressure under control (74.4% vs. 35.2%, *P* <  0.001).

### Urban and rural participants’ HRQL and self-perceptions of aging

Tables 2 and 3 present the results of urban and rural participants’ self-rated HRQL and perceptions of aging. There were statistical differences in all of the dimension scales of HRQL between urban and rural participants except “general health” dimension. In total, urban participants had higher PCS (40.0 ± 12.1 vs. 30.9 ± 8.9, *P* <  0.001) and MCS (51.5 ± 8.3 vs. 46.0 ± 7.8, *P* <  0.001) scores than rural participants. Urban participants’ scores on dimensions of “timeline cyclical”, “consequences negative”, and “control negative” of self-perceptions of aging scale were lower than those of rural participants (*P* <  0.001, respectively), while the scores on dimensions of “consequences positive” and “control positive” were higher (*P* <  0.001, respectively).

### Association between self-perceptions of aging and HRQL

The results of multivariate linear regression for the 7 dimensions scores of the self-perceptions of aging scale and the PCS and MCS scores adjusted for socio-demographic and clinical characteristics for urban and rural participants are presented in Tables 4 and 5. Urban and rural participants who had higher scores on “emotional representations” dimension of self-perceptions of aging had lower PCS and MCS scores. Besides “emotional representations”, for urban participants, “control positive” was the only dimension associated with both PCS and MCS; for rural participants, “timeline chronic” was the only dimension associated with both PCS and MCS. Urban participants' PCS was also associated with “consequences negative”.

## Discussion

### Urban and rural participants’ HRQL

By comparing with general Chinese older people reported in the literature [31], hypertensive older clients in this study had poor HRQL, and the rural participants had poorer physical and mental HRQL than those residing in urban settings. This finding agrees with the study conducted in Shanxi Province, China, which found that urban hypertensive adults had higher HRQL than those in rural areas [32]. More physical/structural and services/assistance barriers [33], and worse economic conditions experienced by rural population may contribute to this difference.

### Urban and rural participants’ self-perceptions of aging

The present study found that older hypertensive rural residents expressed more negative self-perceptions of aging. This is consistent with the study by Li X et al. [13] who found that the elderly who lived in rural areas had lower expectations regarding aging than those in urban areas. Differences in culture and ethical values between rural and urban areas caused by disparities in economic and educational levels may account for the discrepancy of aging perceptions. This is in accordance with the study conducted in Southern Italy, which found that social, environmental, and economic imparities between metropolitan and rural areas determined the difference in aging perceptions among elders aged 75 years or more [34].

### The association between self-perceptions of aging and HRQL among urban and rural participants

Multivariate regression analysis showed that the dimension of “emotional representations” of APQ was associated with both urban and rural participants’ physical and mental HRQL. Individuals who had strong negative emotional responses to aging were inclined to functional disability and depression, resulting in late life maladaptive outcomes [35, 36].

The findings of this study are fairly consistent with those of previous studies demonstrating quality of life as a function of self-perceptions of aging [10, 13]. Possible mediating roles of self-perceptions of aging in the relationship between a poor health condition and quality of life have also been suggested in some studies [6, 37]. Self-perceptions of aging were suggested to be a mediator or moderator of relationships between the subjective health of older adults and quality of life in 20 countries [6] and those between comorbidity and quality of life of older home care clients [37].

### The differences in the association between self-perceptions of aging and HRQL among urban and rural participants

Multivariate regression analysis showed that aging perceptions dimensions which were associated with urban or rural participants’ HRQL were different. For urban hypertensive elders, besides “emotional representations”, “control positive” was the more prominent one which was associated with both physical and mental HRQL, with more positive control indicating better HRQL. According to reports in the literature, older hypertensive patients who had a positive sense of control over their health tended to control hypertension through their own efforts, such as lifestyle changes [10], diet modification [38], as well as medication adherence, which has been demonstrated among urban older hypertensive clients in our previous study [39]. Urban participants’ physical HRQL was also associated with “consequences negative”. High “consequences negative” perception may cause older adults lose confidence in their abilities, resulting in a gradual conscious or unconscious withdrawal in engagement from activities [40]. Different from urban hypertensive elders, rural participants’ physical and mental HRQL were negatively associated with “timeline chronic” dimension. Chronic timeline perception makes older adults always classify themselves as old, tend to explain physical problems in terms of an age attribution, rather than an extenuating circumstance, thus delaying or unwilling to seek treatment for health problems [41].

### Clinical relevance

The findings of this study imply a possibility that the promotion of positive self-perceptions of aging will improve the HRQL of older hypertensive patients. Given the results of our study showing that the dimensions of self-perceptions of aging associated with rural or urban older hypertensive patients’ physical or mental HRQL were different, targeted intervention programs could be designed for rural and urban hypertensive elders to improve their physical and mental HRQL. These interventions may include providing information about positive aspects in old age and false stereotypes about old adults, finding arguments against the negative view on aging, and strengthening the positive view on aging through opinion expression, group discussion, quiz, homework and other techniques based on cognitive behavioral therapy principles. Some researchers have confirmed that the implementation of a self-perceptions of aging intervention could improve mental health of the elderly [42]. Considering the poorer aging perceptions and HRQL, and the larger population of rural elders [43], more measures are warranted among these people.

### Limitations

There are some limitations to this study. First, the sampling was not stratified randomized; therefore, the participants in the study may not adequately represent older hypertensive clients. Moreover, given this study took place in limited regions of China, generalizability of the findings to other countries cannot be guaranteed. Second, the relationship between self-perceptions of aging and HRQL was estimated according to a cross-sectional design; it is difficult to establish the causal association even controlling for the confounders. Third, although the total scale of the Chinese version APQ used in this study had acceptable internal consistency reliability, the reliability of the “emotional representations” subscale was low, with the Cronbach alpha coefficient less than 0.7.

## Conclusions

HRQL was related to self-perceptions of aging in Chinese older hypertensive clients. Rural or urban older adults’ HRQL was associated with different self-perceptions of aging dimensions. Health care professionals should not only control older patients’ blood pressure but also pay attention to their HRQL and self-perceptions of aging, and develop targeted intervention programs for rural and urban older hypertensive people to improve their HRQL.

## Data Availability

Please contact corresponding author for data requests.

## Abbreviations

HRQL: Health-related quality of life
BP: Blood pressure
BMI: Body mass index
SBP: Systolic blood pressure
DBP: Diastolic blood pressure
PF: Physical functioning
RP: Role limitations due to physical problems
BP: Bodily pain
GH: General health perceptions
VT: Vitality
SF: Social functioning
RE: Role limitations due to emotional problems
MH: Mental health
PCS: Physical component summary
MCS: Mental component summary
APQ: Aging perceptions questionnaire
SD: Standard deviation
CI: Confidence interval
